# Progressive increase in vascular access blood flow after percutaneous transluminal angioplasty in patients on hemodialysis

**DOI:** 10.1097/MD.0000000000038408

**Published:** 2024-05-31

**Authors:** Hyun Jin Koh, Seung-Jung Kim, Shina Lee

**Affiliations:** a Department of Internal Medicine, School of Medicine, Ewha Womans University, Seoul, Korea.

**Keywords:** angioplasty, arteriovenous shunt, blood flow velocity, dialysis

## Abstract

Percutaneous transluminal angioplasty (PTA) is the gold standard for treating stenoses with dysfunctional vascular access. Recently, we found that vascular access blood flow (VABF) measured immediately after PTA increased over time without the need for additional procedures in the patients who underwent PTA. Therefore, this study was conducted to confirm an increase in VABF after PTA and identify the factors associated with it. Patients on chronic hemodialysis at a single institution were retrospectively reviewed and those with accesses that had a measurement of VABF immediately after PTA and within 1 month from PTA were included in the study. The relationship between clinical parameters and changes in VABF were analyzed using paired t-test and linear regression. A total of 47 PTA accesses (fistulas, 26; grafts,21) were included. The mean VABF on the day of PTA and the following measurement were 796.9 ± 329.1 mL/min and 1105.1 ± 410.3 mL/min, respectively. In the univariate analysis, the diameter of the balloon catheter used in the PTA and serum uric acid (SUA) level were significantly associated with an increase in VABF. Atrial fibrillation was a significant factor for the percentage change in vascular access. In the multivariate analysis, SUA level, balloon catheter diameter, and atrial fibrillation remained independent factors for changes in VABF and percentage change in VABF, respectively. The study identified progressive increases in the VABF after PTA without additional procedures. SUA level, balloon catheter diameter used in PTA, and atrial fibrillation were independently associated with changes in VABF.

## 1. Introduction

Successful maintenance of vascular access is a key factor in the quality and longevity of life of patients with end-stage kidney disease (ESKD) receiving hemodialysis. However, many patients experience vascular access dysfunction, such as stenotic or thrombosed arteriovenous fistulas (AVF) and arteriovenous grafts (AVG). Percutaneous transluminal angioplasty (PTA) has become the gold standard treatment for venous stenosis associated with AVFs and AVGs with excellent success rates.^[1]^

The K/DOQI guidelines state that a PTA-treated lesion should return to acceptable limits of the clinical/physiological parameters used to detect the stenosis as well as restore anatomical defects with the residual stenosis being <30% in the subsequent angiography.^[2]^ Clinically, regular vascular access flow monitoring has been performed to evaluate access adequacy immediately after PTA, although there is insufficient evidence to recommend routine surveillance imaging.^[3]^ In our institution, monthly access flow monitoring is conducted; therefore, the access flow was re-measured within a month from the date of PTA. Interestingly, an increased vascular access flow rate was observed between the day of PTA and subsequent measurements within 30 days without additional procedures.

Hence, the present study aimed to confirm the progressive increase in vascular access blood flow (VABF) after PTA without additional procedures and identify the factors associated with it.

## 2. Materials and methods

### 2.1. Study participants

The electronic medical records of patients who underwent chronic hemodialysis at a single institution between March 2018 and January 2020 were retrospectively reviewed. A total of 264 patients, who underwent maintenance hemodialysis for more than 3 months, were eligible for the study. The cohort included patients with permanent subcutaneous access to either native AVFs or synthetic grafts (AVGs); however, patients with catheters were excluded. Since 2011, routine monthly access flow monitoring was initiated in our institution according to the K/DOQI guideline to detect stenosis and determine the progress of PTA. When this surveillance, using ultrasound dilution technique, showed a decline in access flow by more than 25% in the past 3 months and one or more prominent clinical indicators, it was considered an indication for PTA. The clinical indicators included: abnormal physical condition on vascular access, elevated venous pressure, difficulty in cannulation, aspiration of clots, and an unexplained decrease in the delivered dialysis dose. The final decision to proceed with PTA depended on the nephrologist who based it on the integrated information of the objective flow rate and clinical indicators.

**Figure 1.**
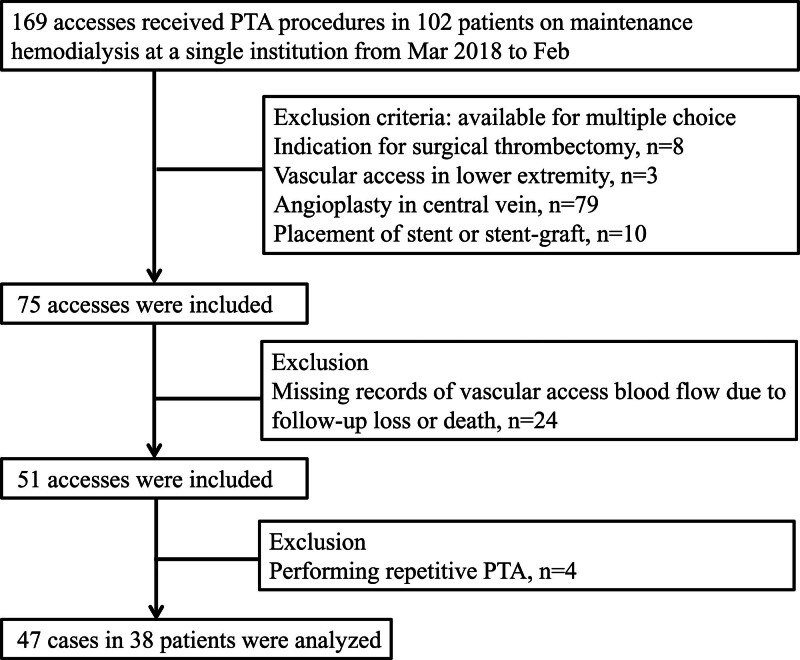
Flow diagram for patient enrollment.

Throughout the retrospective chart review, 169 accesses located in the upper extremities that underwent PTA within the study period were initially evaluated for eligibility. The exclusion criteria were: indication for surgical thrombectomy, placement of a stent or stent-graft, performance of angioplasty in the central vein, confirmation of elastic recoil, missing records of access flow after PTA, and repeated PTA within 1 month from the previous PTA. Since the study was conducted on patients undergoing maintenance hemodialysis for more than 3 months, the vascular accesses were considered to be fully mature. Finally, 47 accesses from 38 patients were included in the study (Fig. 1). Overlapping patients have undergone PTA with a minimum interval of 3 months. The study protocol was approved by the Institutional Review Board. Informed consent was waived due to the retrospective nature of the study and the use of anonymous clinical data. All clinical investigations were conducted in accordance with the ethical standards of the 1964 Declaration of Helsinki and its subsequent amendments.

### 2.2. VABF measurement technique

Hemodialysis VABF was measured using the ultrasound velocity dilution technique with a Transonic HD01 hemodialysis monitor (Transonic Systems, Inc., Ithaca, NY). This technique has been extensively validated.^[4,5]^ In summary, 1 hour after the initiation of dialysis, the system employs 2 ultrasonic sensors affixed to the catheters of the hemodialysis tubing - one on the arterial and another on the venous catheter. These sensors are positioned approximately 2 to 6 inches away from the connection point of the tubing to the dialysis needles. Initially, blood recirculation was checked in the vascular access while the blood catheters were in the normal position. Then the blood catheters were reversed, and ultrafiltration was turned off. The blood pump flow rate was set to 250 mL/min and a measured bolus of saline (10 mL) was released into the venous catheter, diluting the flow of blood in the access area and resulting in changes in sound velocity that were measured by the transducers on the catheters. This change was then calculated using Transonic software, yielding the VABF in milliliters per minute.

### 2.3. PTA procedure

Previously reported PTA procedures and techniques were used in this study. Briefly, under fluoroscopy, a 6 to 7 Fr catheter introducer sheath was inserted toward the stenosis site using a 20-gauge needle. After administration of 3000 units of heparin intravenously, PTA was usually performed with a noncompliant balloon of 4 to 7 mm in diameter (in some patients, semi-compliant balloons were used), usually multiple times, with 30 seconds to 3 minutes of expansion each time, with the goal of complete balloon expansion and achieving 30% or less residual stenosis. In thrombosed AVFs or AVGs, an additional 2000–3000 IU of heparin was administered through the thrombotic segment, and thrombectomy was performed to remove the thrombi. The decluttering procedure was performed using a 7F Desilets-Hoffman sheath (Cook, Bloomington, IN) and a 5 Fr non-over-the-wire Fogarty thrombectomy balloon catheter (Edwards Life Sciences, Irvine, CA) to mechanically aspirate the thrombus. The treatment endpoint was either complete balloon expansion or the burst pressure rate during inflation. The balloon diameter was determined by measuring the vein diameter before and after stenosis, using ultrasonography or other imaging modalities. Immediately after the procedure, whole AVF angiography from the feeding artery to the superior vena cava was performed to treat residual stenosis or adherent thrombosis, as needed. After PTA, the introducer sheath of the catheter was removed, and immediate hemostasis was achieved.

### 2.4. Laboratory test

Laboratory data was obtained during routine checkups at the beginning of each month. Venous blood was sampled immediately before starting the dialysis session after 8 hours of fasting. Routine laboratory methods were used to measure blood hematocrit, albumin, total calcium, phosphate, and uric acid levels.

### 2.5. Statistical analysis

Categorical variables are expressed as numbers (N) and percentages, while continuous variables are expressed as means and standard deviations (SDs). The differences in pairing variables, including access flow, actual blood pump flow, arterial line pressure, venous line pressure, ultrafiltration, and blood pressure between the measurements on the day of PTA and the subsequent routine surveillance were made using paired t-tests. Univariate regression analyses were used to evaluate the predictors of increased VABF, and the enter method of multiple regression was used to adjust for covariates identified by univariate analyses and clinically relevant variables. Results of regression analyses were presented as β with corresponding 95% confidence intervals (CIs) and *P* values. All *P* values were 2-sided, and a *P* value <.05 was considered statistically significant. All statistical analyses were performed using IBM SPSS (version 18.0; SPSS Inc., Chicago, IL).

## 3. Results

### 3.1. Baseline characteristics

The baseline demographics and clinical characteristics of the patients are shown in Table 1. A total of 47 accesses underwent PTA and the VABF increased in all accesses. The mean age of access was 27.9 ± 19.3 months and the median time for access flow measurement interval between PTA and the following routine surveillance measurement was 18.6 ± 9.0 days. Fifty-five percent of the accesses had AVFs, the remaining had AVGs, with 74% of the accesses being located on the forearm. Twenty-five accesses (55.3%) had a history of permanent hemodialysis catheter insertion, and 6 of the catheters had been placed on the ipsilateral side of AVF or AVG creation. The vascular accesses were occluded in 23.4% of enrolled accesses at the time of PTA, and the stenotic lesions were located on outflow (66%), inflow (29.8%), and both (4.3%), in order of frequency. The diameter of the balloon catheter used during the PTA procedure was 6, 7, and 8 mm, with an average diameter of 6.4 ± 1.0 mm. The mean age of the enrolled patients was 67.2 ± 12.4 years old, with 20 male and 27 female patients. All patients had hypertension and were taking medications, specifically either angiotensin-converting enzyme inhibitors or angiotensin II receptor blockers. Additionally, 63% of the cohort were undergoing treatment for diabetes mellitus. Thirty-three percent of the subjects showed ejection fractions <45% and 9% of patients were diagnosed with atrial fibrillation and were taking anticoagulants. None of the patients were prescribed uric acid-lowering drugs.

**Table 1:** Demographics and clinical characteristics.

Variables	Mean ± SD
Male/Female, n	20/27
Age (yr)	67.2 ± 12.4
Comorbidity	
Diabetes, n (%)	30 (63.8)
Hypertension, n (%)	47 (100)
Ejection fraction < 45%, n (%)	15 (31.9)
Atrial fibrillation, n (%)	5 (10.6)
Access age (mo)	27.9 ± 19.3
History of perm catheter insertion, n (%)	25 (53.1)
Ipsilateral side insertion, n (%)	6 (12.7)
Access type	
Fistula, n (%)	26 (55.3)
Graft, n (%)	21 (44.7)
Upper arm, n (%)	12 (25.5)
Forearm, n (%)	35 (74.5)
Median time interval between the day of PTA and the day of following measurement of routine surveillance (days)	18.6 ± 9.0 (3–29)
PTA site	
Inflow, n (%)	14 (29.8)
Outflow, n (%)	31 (66.0)
Both, n (%)	2 (4.3)
Reason for PTA, occlusion (n, %)	11 (23.4)
Balloon diameter (mm)	6.4 ± 1.0
Laboratory findings	
Hematocrit (%)	31.0 ± 3.5
Albumin (g/dL)	4.0 ± 0.4
Total calcium (mg/dL)	8.6 ± 0.7
Phosphorus (mg/dL)	4.7 ± 1.1
Uric acid (mg/dL)	6.0 ± 1.6

PTA = percutaneous transluminal angioplasty, SD = standard deviation.

### 3.2. Comparisons of VABF and the related parameters between after immediately after PTA and following measurement

The mean VABF was 796.9 ± 329.1 mL/min and 1105.1 ± 410.3 mL/min immediately after PTA and on the subsequent routine surveillance, respectively. Moreover, during hemodialysis, both the mean actual blood pump flow and the absolute arterial and venous line pressures were significantly increased between these time points. At each hemodialysis session, blood pressure, including systolic, diastolic, and mean arterial pressure, showed insignificant differences, although the amount of ultrafiltration was significantly higher on the day of routine surveillance than immediately after PTA.

Next, we investigated whether the covariants including dialysis prescription and blood pressure mentioned above were associated with an increase in VABF between the time point immediately after PTA and the following measurement within a month using a regression model. The changes in VABF were expressed as the absolute difference in vascular access between the time points and the percentage change in VABF of the following measurement to the VABF measurement on the day of PTA. Although the dialysis prescription factors showed significant differences at each dialysis session, the actual blood pump flow, arterial line pressure, and venous line pressure were not associated with changes in either vascular access flow or the percentage change in vascular access flow between the time points of dialysis. Moreover, the change in VABF was not related to the amount of ultrafiltration or blood pressure, including systolic, diastolic, and mean arterial pressures (Table 2).

**Table 2:** Univariate regression of factors related hemodialysis prescription for the change of vascular access blood flow.

Change of measurements	Change of vascular access (mL/min)			Percentage change of vascular access (%)		
	β coefficient	95 % CI	*P* value	β coefficient	95 % CI	*P* value
Δ Actual blood pump flow, mL/min	−0.3	−3.8 to 3.2	.863	−0.1	−0.6 to 0.5	.803
Δ Arterial line pressure, mm Hg	2.0	−1.2 to 5.2	.207	0.3	−0.2 to 0.8	.219
Δ Venous line pressure, mm Hg	−1.7	−5.2 to 1.7	.309	−0.1	−0.7 to 0.4	.686
Δ Systolic blood pressure, mm Hg	0.0	−5.8 to 5.7	.988	0.4	−0.5 to 1.3	.358
Δ Diastolic blood pressure, mm Hg	2.8	−7.9 to 13.5	.596	0.5	−1.2 to 2.1	.578
Δ Pulse rate per min	2.2	−8.7 to 13.1	.688	0.0	−1.8 to 1.7	.963
Δ Mean arterial pressure, mm Hg	3.0	−6.3 to 12.3	.523	0.8	−0.7 to 2.2	.284
Δ Amount of fluid removed during a dialysis session (kg)	4.9	−87.0 to 96.8	.915	0.2	−14.2 to 14.6	.982

The patient characteristics, including sex, age, presence of diabetes mellitus, atrial fibrillation, low ejection fraction (<45%), history of permanent catheter insertion, and laboratory findings of hematocrit, serum albumin, total calcium, phosphorus, and uric acid levels, were also explored to identify the factors associated with increased VABF. The level of uric acid was significantly associated with the absolute change in VABF (β coefficient: −54.0, 95 % CI: −95.9 to −12.0, *P* = .013), and comorbidity of atrial fibrillation was associated with the percentage change in VABF (β coefficient: 94.5, 95 % CI: 43.7–145.39, *P* value = .001), shown in Table 3. In addition, factors related to vascular access, such as access age, graft vs fistula, placement of vascular access (forearm vs upper arm), location of the stenotic lesion (outflow vs inflow), occlusion of vascular access, and balloon catheter diameter used during the procedure were examined. The results suggest that the diameter of the balloon catheter was significantly associated with the absolute change in VABF (β coefficient: 89.6, 95 % CI: 26.3–152.9, *P* value = .007).

**Table 3:** Univariate regression analysis for change of vascular access blood flow.

	Change of vascular access (mL/min)			Percentage change of vascular access (%)		
	β coefficient	95 % CI	*P* value	β coefficient	95 % CI	*P* value
Female	−4.8	−148.5 to 138.9	.946	11.8	−10.5 to 34.0	.291
age	−0.5	−6.2 to 5.3	.874	0.1	−0.8 to 1.0	.895
Diabetes	6.3	−143.0 to 155.7	.932	22.1	−0.3 to 44.5	.053
Ejection fraction < 45%	−14.3	−163.6 to 134.9	.847	0.6	−22.8 to 24.0	.960
Atrial fibrillation	161.1	−81.3 to 403.5	.187	94.5	43.7–145.39	.001
History of permanent catheter insertion	31.0	−110.4 to 172.4	.661	−6.6	−28.7 to 15.6	.553
History of Ipsilateral catheter insertion	111.7	−92.6 to 315.9	.276	−1.1	−33.6 to 31.4	.945
Hematocrit (%)	−0.3	−20.9 to 20.4	.980	−0.2	−3.4 to 3.0	.900
Albumin (g/dL)	21.7	−154.2 to 197.6	.805	2.2	−25.4 to 29.8	.871
Total calcium (mg/dL)	30.1	−64.4 to 124.6	.524	6.9	−7.9 to 21.6	.352
Phosphorus (mg/dL)	−12.5	−75.0 to 49.9	.688	−2.9	−12.7 to 6.9	.551
Uric acid (mg/dL)	−54.0	−95.9 to −12.0	.013	−4.4	−11.4 to 2.5	.207
Access age per 1 mo	2.0	−1.6 to 5.7	.265	0.1	−0.8 to 1.0	.895
Graft vs fistula	82.8	−56.0 to 221.6	.236	−13.6	−56.1 to 28.7	.519
Upper arm vs forearm	−2.6	−34.0 to 28.8	.867	1.5	−3.4 to 6.4	.528
Stenotic lesion, outflow vs inflow	59.7	−66.8 to 186.3	.346	4.6	−18.9 to 28.0	.696
Occlusion of vascular access	29.3	−139.8 to 198.4	.729	1.4	−25.2 to 27.9	.917
Balloon diameter (mm)	89.6	26.3–152.9	.007	16.3	−2.04 to 34.64	.080

In clinical practice, assuming that patients characteristics and vascular access parameters were given equal importance, the factors of dialysis prescription, blood pressure, number of the days between the time point of the vascular access measurement, access age, access type (graft vs fistula), placement of access (upper vs lower arm), location of stenotic lesion (inflow vs outflow), and occurrence of access occlusion, age, sex, comorbidities of diabetes, low ejection fraction (<45%), and atrial fibrillation were selected for adjustment (model 1, adjusted for change of actual blood pump flow, arterial line pressure, venous line pressure, systolic blood pressure, diastolic pressure, number of days between 2 measurements; model 2, additional adjusted for access age, access type (graft and fistula), location of access (upper vs lower arm), location of stenosis lesion (inflow vs outflow), and presence of access occlusion on model 1; model 3, additional adjusted for age, sex, history of diabetes, ejection fraction (<45%) or atrial fibrillation for percentage of VABF on Model 2). Analysis of these results as shown in Table 4 demonstrated that serum uric acid was associated to absolute change of VABF independently (Model 1, β coefficient: −72.4, 95% CI: −120.2 to − 24.7, *P* value = .004; Model 2, β coefficient: −95.4, 95% CI: −153.2 to −37.6, *P* value = .024; Model 3, β coefficient: −98.2, 95% CI: −161.1 to −35.5, *P* value = .003). The balloon catheter diameter was significantly associated with changes in vascular access flow when adjusted for dialysis prescription, blood pressure, access type, access location, stenosis, and occlusion (Models 1 and 2), although the independent effect of balloon catheter diameter on the change in VABF was weakened when adjusted for additional demographic characteristics (Model 3).

**Table 4:** Multivariate regression for the change of vascular access blood flow.

	Change of vascular access (mL/min)					
	Model 1		Model 2		Model 3	
	β coefficient (95% CI)	*P* value	β coefficient (95% CI)	*P* value	β coefficient (95% CI)	*P* value
Uric acid, per 1 mg/dL increase	−72.4 (−120.2 to −24.7)	.004	−95.4 (−153.2 to −37.6)	.024	−98.2 (−161.1 to −35.5)	.003
Balloon catheter diameter, per 1 mm increase	69.6 (3.1–136.1)	.041	99.9 (12.5–187.4)	.013	83.8 (−15.4 to 183.1)	.095

Model 1: adjusted for change of actual blood pump flow, arterial line pressure, venous line pressure, amount of fluid removed during a dialysis session, systolic blood pressure, diastolic pressure, number of days between 2 measurements.

Model 2: additional adjusted for access age, access type (graft and fistula), location of access (upper vs lower arm), location of stenosis lesion (inflow vs outflow), and occurrence of access occlusion on model 1.

Model 3: additional adjusted for age, sex, history of diabetes, ejection fraction <45%, atrial fibrillation on Model 2.

With regard to percentage change in vascular access, atrial fibrillation remained an independent factor (model 1: β coefficient: 89.7, 95% CI: 33.5–145.8, *P* value = .003; model 2: β coefficient: 78.3, 95% CI: 8.93–147.8, *P* value = .028) unless additional adjustment for demographic characteristics on model 2 were made, which was adjusted for multiple covariants including dialysis prescription, access status, and factors about PTA (Table 5).

**Table 5:** Multivariate regression analysis for the percentage change of vascular access blood flow.

	Percentage change of vascular access (%)					
	Model 1		Model 2		Model 3	
	β coefficient (95% CI)	*P* value	β coefficient (95% CI)	*P* value	β coefficient (95% CI)	*P* value
Presence of atrial fibrillation	89.7 (33.5–145.8)	.003	78.3 (8.93–147.8)	.028	97.8 (−35.84 to 231.10)	.145

Model 1: adjusted for change of actual blood pump flow, arterial line pressure, venous line pressure, amount of fluid removed during a dialysis session, systolic blood pressure, diastolic pressure, number of days between 2 measurements.

Model 2: additional adjusted for access age, access type (graft and fistula), location of access (upper vs lower arm), location of stenosis lesion (inflow vs outflow), and occurrence of access occlusion on model 1.

Model 3: additional adjusted for age, sex, history of diabetes, ejection fraction <45% on Model 2.

## 4. Discussion

There have been few reports of progressive increases in the access blood flow rate after PTA. In the present study, an increased access blood flow rate was demonstrated between immediately after PTA and the routine survey within 30 days after PTA without additional procedures. The novelty of our investigation lies in its focus on the progressive increase in vascular access flow after PTA. The observed rise in VABF suggests that careful consideration is necessary when planning for repeated PTA. If the measured VABF immediately after PTA is lower than expected, repeated VABF measurements become essential for planning the next PTA unless vascular access is deemed insufficient for dialysis. Moreover, independent associations were identified between changes in vascular access flow rate and the serum uric acid level, the diameter of the balloon catheter used in PTA, and the comorbidity of atrial fibrillation. These findings highlight additional factors that should be taken into account when assessing and managing vascular access flow rates.

In the present study, the mean arterial pressure, systolic blood pressure, and diastolic blood pressure were not significantly different immediately after PTA and in the following the routine survey (Table 6). Consequently, it was postulated that the impact of blood pressure on changes in VABF was minimal or nonexistent. Furthermore, cardiac output has been reported to decrease more than 10% after intermittent hemodialysis and/or ultrafiltration in multiple studies in patients with stable ESKD with or without a propensity for intradialytic hypotension.^[6–8]^ This reduction is attributed to not to ultrafiltration itself but to the changes induced by ultrafiltration-induced changes in blood pressure, pulse pressure, pulse rate, and blood volume, collectively leading to a decrease in cardiac.^[9–13]^ Despite the larger amount of ultrafiltration at the time of the routine measurement compared to immediately after PTA, as shown in the present study, there was an increased in VABF in the following the routine survey.

**Table 6:** Comparisons of vascular access blood flow and the related parameters between immediately after PTA and the following measurement of routine surveillance.

Parameters (Mean ± SD)	Immediately after PTA	Following measurement	*P* value
VABF (mL/min)	796.9 ± 329.1	1105.1 ± 410.3	<.001
Actual blood pump flow, mL/min	253.6 ± 20.4	263.8 ± 23.8	.002
Arterial line pressure, mm Hg	−98.60 ± 36.0	−114.71 ± 37.7	.003
Venous line pressure, mm Hg	119.1 ± 26.1	129.6 ± 27.4	.001
SBP, mm Hg	143.5 ± 24.6	141.7 ± 21.5	.538
DBP, mm Hg	72.7 ± 13.0	72.7 ± 12.8	.968
Mean arterial pressure	67.3 ± 13.3	93.5 ± 12.3	.837
Pulse rate per min	74.8 ± 12.6	73.3 ± 12.9	.274
Amount of fluid removed during a dialysis session (kg)	2.1 ± 1.1	2.4 ± 1.1	.022

DBP = diastolic blood pressure, PTA = percutaneous transluminal angioplasty, SBP = systolic blood pressure, SD = standard deviation, VABF = vascular access blood flow.

It is well known that the creation of vascular access decreases total peripheral resistance, by adding vascular access resistance in parallel with systemic peripheral resistance, and significantly increases cardiac output.^[14–17]^ Numerous studies have suggested a proportional relationship between vascular access flow and cardiac output. Basile et al proposed a complex relationship rather than a linear correlation between these 2, which consisted of an initial plateau followed by a steep slope.^[16]^ Lopot et al reported a more than 2-fold increase in vascular access flow after PTA, with practically unchanged cardiac output 1 week after PTA.^[18]^ Similarly, no immediate change in cardiac output was observed 5 days after the surgical vascular access bandage decreased vascular access flow.^[18]^ Another study reported that both vascular access flow volume and cardiac output were increased by PTA in most cases, whereas in some cases, the cardiac output decreased despite an increase in vascular access flow volume.^[19]^ Beyond its association with cardiac output, baseline VABF serves as the most reliable predictor for changes in VABF post-PTA. Stenosis minimum luminal diameter cutoffs of ≤ 2.0 and > 4.5 mm are valuable in forecasting the extent of changes in VABF.^[20]^ The diversity in study characteristics and variations in methodologies and temporal parameters for measuring VABF and cardiac output make it challenging to draw definitive conclusions. Regardless of whether cardiac output increased after PTA, these studies and our findings suggest the necessity of shifting the perspective from the physiological response of the vascular circuit to localized microvascular dysfunction to investigate progressive increases in vascular access flow after PTA.

About 10 years after the first report of angioplasty for AVF stenosis in 1981,^[21]^ Davidson et al demonstrated the mechanisms of successful PTA using an intravascular ultrasound.^[22]^ They provided morphological information following PTA, in which vessel stretching, dissection, and elastic recoil were noted in order of prevalence. Vessel stretching and dissection are similar to the mechanisms validated by histological studies of coronary artery balloon angioplasty. A couple of studies have shown that histological sections of human arteries after successful PTA show the splitting of atheromatous plaques. The split extended to the internal elastic membrane. As the angioplasty balloon becomes fully inflated, the elastic media and adventitia stretch to conform to the outer diameter of the expanded balloon.^[23,24]^ An animal study using atherosclerotic rabbit femoral arteries showed that PTA caused radial stretching of the artery, medial compression, intramural hemorrhage, injury to normal arterial segments, and dissection of the intima and media.^[25]^ Notably, in this study, the minimal luminal diameter (MLD) increased immediately after PTA, whereas a rapid decrease in MLD was noted between days 1 and 3, followed by a slower decrease between days 3 and 14. The MLD at 28 days increased over the 14-day MLD and approximated the post-angioplasty MLD. The decrease in MLD during the specific period, in the beginning, may be explained as elastic recoil of the stretched normal segment and thrombus formation within the dissection in the early period of 1 to 3 days, followed by smooth muscle cell proliferation, and increased extracellular matrix formation in the late period of 14 to 28 days. This study could be implicated in our findings as an increase in VABF several days after PTA. The principles of Poiseuille law, consisting of vascular radius and resistance, do not directly apply to the AVF model; however, there is a definite relationship between the effects of PTA-induced decreased vascular resistance, increased diameter, and blood flow.^[26]^ For example, a 2-fold increase in vessel diameter decreases resistance 16-fold, and in turn, the decreased resistance produces an exponential increase in vascular access flow. This premise, based on Poiseuille law, is applicable to our finding that the size of the balloon catheter is associated with an increase in VABF. However, the mechanisms underlying the significant increase in vascular blood flow between immediately after PTA and several days after PTA are unclear because of the lack of studies on human arteriovenous shunts; nonetheless, there may be a difference in response to PTA-induced vascular injury within an arterialized vein which may result in a progressive increase in VABF.

Elastic recoil occurs most frequently when treating central venous lesions. It was reported that venous elastic recoil occurred as recurrent luminal narrowing of >50% within 15 minutes after full effacement of angioplasty.^[27]^ By 15 minutes, 15.6% of treated lesions recurrently narrowed in more than half of cases, with the majority observed at 5 minutes (63%). However, elastic recoil did not influence patency, with 34.8% of 6-month primary patency in grafts and 47.1% in fistulas. To reduce confusion from the elastic recoil-induced decreased blood flow, cases of PTA in the central vein were excluded, and the absence of elastic recoil in the included cases was confirmed by reviewing the full clips of the PTA procedure.

The independent associations between changes in VABF and serum uric acid, and between the percentage change in VABF and the presence of atrial fibrillation were significant findings in our study (Table 3). PTA inevitably causes vascular injuries. Endothelial denudation is considered a primary injury after balloon angioplasty and/or stent implantation. With the denudation of a small area of the endothelial surface, little to no intimal hyperplasia was observed, whereas when larger areas were denuded, there was a greater degree of intimal thickening.^[28–30]^ In addition, more severe and deeper injuries to the vessel wall result in delayed re-endothelization.^[31]^ In porcine coronary arteries with stenting, the regenerating endothelium experiences long-term dysfunction for more than 3 months, characterized by impaired endothelium-dependent vasodilatation.^[32]^ PTA-induced endothelial denudation and subsequent endothelial dysfunction may hinder vascular dilatation immediately after PTA; however, vessel stretching by PTA itself is sufficient to redeem the negative effect of vascular injury on VABF. Both uric acid and atrial fibrillation are well-recognized contributors of endothelial dysfunction,^[33,34]^ but the β coefficient as shown in Tables 3 and 4, had a positive value for atrial fibrillation and a negative value for uric acid resulting in an opposite effect on the change of VABF. More specifically, atrial fibrillation is associated with a decrease in ventricular filling and cardiac preload, resulting in reduced cardiac output, which might contribute to the decrease in VABF. Although it is unclear how both factors contribute to the transition from endothelial dysfunction to regeneration after PTA, advanced chronic kidney disease is the strongest risk factor for endothelial dysfunction beyond other risk factors.^[35]^ Thus, the effects of both uric acid and atrial fibrillation on changes in VABF may have significant clinical implications.

This study had several limitations. The major limitations were the retrospective design from a single institution, absence of a control group, and relatively small number of subjects. Additionally, patients with missing data were excluded which may have caused a loss of information. Therefore, there may be a selection bias originating from the poorly defined inclusion and exclusion criteria. Furthermore, more than half of the patients who required surgical thrombectomy, stent or stent-graft placement, and repetitive PTA were excluded from the study population which might have limited the conclusions. In addition, ambiguously defined covariants may have yielded skewed interpretations of the results. Specifically, pathophysiological differences in neointimal formation and PTA mechanisms between AVF and AVG were not considered, and the recruited accesses were limited to the upper extremities. Finally, the laboratory parameters did not reflect the patient condition on each day of PTA because the laboratory data was obtained from routine monthly checkups. Therefore, well-designed studies are needed to verify these results.

## 5. Conclusion

In successful PTA, VABF increased consecutively in the early period after PTA. Moreover, serum uric acid level, balloon catheter diameter used in PTA, and atrial fibrillation were independently associated with changes in VABF. Further studies are required to verify our results and identify the effect of progressive increases in VABF on vascular access patency after PTA.

## Author contributions

**Conceptualization:** Hyun Jin Koh, Seung-Jung Kim, Shina Lee.

**Data curation:** Hyun Jin Koh, Shina Lee.

**Formal analysis:** Seung-Jung Kim, Shina Lee.

**Methodology:** Shina Lee.

**Supervision:** Seung-Jung Kim, Shina Lee.

**Validation:** Shina Lee.

**Visualization:** Shina Lee.

**Writing – original draft:** Shina Lee.

**Writing – review & editing:** Hyun Jin Koh, Seung-Jung Kim, Shina Lee.
